# Improved delivery of Cas9 protein/gRNA complexes using lipofectamine CRISPRMAX

**DOI:** 10.1007/s10529-016-2064-9

**Published:** 2016-02-18

**Authors:** Xin Yu, Xiquan Liang, Huimin Xie, Shantanu Kumar, Namritha Ravinder, Jason Potter, Xavier de Mollerat du Jeu, Jonathan D. Chesnut

**Affiliations:** Synthetic Biology Department, Thermo Fisher Scientific, 5781 Van Allen Way, Carlsbad, CA 92008 USA

**Keywords:** Cas9 protein, Cell engineering, CRISPR, CRISPRMAX, Genome editing, Homologous recombination

## Abstract

**Objectives:**

To identify the best lipid nanoparticles for delivery of purified Cas9 protein and gRNA complexes (Cas9 RNPs) into mammalian cells and to establish the optimal conditions for transfection.

**Results:**

Using a systematic approach, we screened 60 transfection reagents using six commonly-used mammalian cell lines and identified a novel transfection reagent (named Lipofectamine CRISPRMAX). Based on statistical analysis, the genome modification efficiencies in Lipofectamine CRISPRMAX-transfected cell lines were 40 or 15 % higher than those in Lipofectamine 3000 or RNAiMAX-transfected cell lines, respectively. Upon optimization of transfection conditions, we observed 85, 75 or 55 % genome editing efficiencies in HEK293FT cells, mouse ES cells, or human iPSCs, respectively. Furthermore, we were able to co-deliver donor DNA with Cas9 RNPs into a disrupted EmGFP stable cell line, resulting in the generation of up to 17 % EmGFP-positive cells.

**Conclusion:**

Lipofectamine CRISPRMAX was characterized as the best lipid nanoparticles for the delivery of Cas9 RNPs into a variety of mammalian cell lines, including mouse ES cells and iPSCs.

**Electronic supplementary material:**

The online version of this article (doi:10.1007/s10529-016-2064-9) contains supplementary material, which is available to authorized users.

## Introduction

The use of cell-penetrating peptides (CPPs) facilitates the delivery of active proteins into cells (Copolovici et al. 2014; Erazo-Oliveras et al. 2014; Jo et al. 2014). The delivery of purified Cas9 protein and gRNA complexes (Cas9 RNPs) has gained increasing attention due to the high cleavage efficiency and potentially lower off-target effect compared with plasmid DNA transfection (Kim et al. 2014; Zuris et al. 2015; Liang et al. 2015). Although Cas9 RNPs can be delivered into mammalian cells via electroporation with relatively high efficiency, lipid-mediated transfection remains popular due to ease of use, low cost, and adaptation to high throughput system. Several methods for delivery of Cas9 RNPs have been reported. Through conjugation of a cell-penetrating peptide to Cas9 protein and complexation of gRNA with CPPs, the subsequent Cas9 RNPs were able to deliver into HEK293T, HeLa, NCCIT (human embryonal carcinoma cell line), HDF (human dermal fibroblasts), and H9 human embryonic stem cells with up to 36 % genome modification efficiency (Ramakrishna et al. 2014). A method termed iTOP (induced transduction by osmocytosis and propanebetaine) allows the highly efficient transduction of native proteins into a wide variety of primary cell types (D’Astolfo et al. 2015). Furthermore, by fusing to negatively supercharged GFP proteins or binding to anionic nucleic acids, functional proteins could be delivered into mammalian cells via cationic lipid-mediated transfection. As described, Cas9 RNPs delivered into U2OS cells using Lipofectamine 2000 resulted in up to 80 % modification efficiency at an integrated GFP reporter and Cas9 RNPs delivered into the mouse inner ear in vivo using Lipofectamine RNAiMAX resulted in 20 % loss of GFP expression in auditory sensory cells (Zuris et al. 2015). However, significant toxicity from Lipofectamine 2000 was also reported in U2OS cells. In the present study, we screened a large set of transfection reagents using relatively easy and hard-to-transfect cell lines and identified a new transfection reagent, Lipofectamine CRISPRMAX, which worked significantly better than Lipofectamine 3000 or Lipofectamine RNAiMAX with very low cell toxicity.

## Methods

### Systematic design of experiments used for screening transfection reagents

The screening of transfection reagents was conducted in a 96-well format. 1 day prior to transfection, six commonly used cell lines, A549, HEK293, HeLa, HepG2, MCF-7, and U2OS, were seeded in 96-well plates at 10,000–20,000 cells per well. On the day of transfection, a master mix of Cas9 protein and HPRT1 gRNA was prepared in Opti-MEM medium and incubated for 5 min at 25 °C to form the Cas9 RNPs. The amount of Cas9 RNPs was held constant at 40 ng GeneArt Platinum Cas9 nuclease and 8.5 ng gRNA per well. On the other hand, the amount of each transfection reagent, which was also prepared in Opti-MEM medium, varied from 0.1, 0.2, 0.4 and 0.6 µl per well. Lipofectamine 3000 and Lipofectamine RNAiMAX served as controls. The Cas9 RNPs in Opti-MEM medium were added to the transfection reagents diluted in Opti-MEM medium. The mixture was incubated at 25 °C for 10–15 min to form the Cas9 RNPs and transfection reagent complexes, followed by addition to the cells. After incubation for 48 h, the cells were lysed and percentage of Indel (insertion and deletion) was measured by GeneArt Genomic Cleavage Detection Kit. The experimental data was then analyzed using JMP, Version 11. SAS Institute Inc. (Cary, NC, USA).

### Cell transfection in a 24-well plate using CRISPRMAX

One day prior to transfection, adherent cells were plated onto 24-well plates at 0.4 to 1.5 × 10^5^ cells per well in 500 µl of growth medium so that the cells reached 30–70 % confluence at the time of transfection. On the day of transfection, 25 µl of Opti-MEM medium was added to a 1.5 ml sterile Eppendorf tube, followed by the addition of 500 ng GeneArt Platinum Cas9 nuclease and 125 ng gRNA. Upon mixing by vortexing briefly, 1 µl Cas9 Plus reagent was added to the solution containing Cas9 protein and gRNA. After briefly vortexing, the mixture was incubated at 25 °C for 5 min to allow the formation of Cas9 RNPs. The Cas9 RNPs remained active at 25 °C for up to 2 h. For co-delivery of donor DNA, 500 ng single strand DNA oligonucleotide or 300 ng linear PCR fragment was added to the Cas9 RNPs at this point. Meanwhile, 25 µl Opti-MEM medium was added to a separate sterile Eppendorf tube, followed by addition of 1.5 µl of Lipofectamine CRISPRMAX. After briefly vortexing, the Lipofectamine CRISPRMAX solution was incubated at 25 °C for approx. 5 min. After incubation, the Cas9 RNPs were then added to the Lipofectamine CRISPRMAX solution. The reverse addition of Lipofectamine CRISPRMAX solution to the Cas9 RNPs was found to decrease the editing efficiency in certain cell lines. Upon mixing, the sample was incubated at 25 °C for 10–15 min to form Cas9 RNPs and Lipofectamine CRISPRMAX complexes and then added to the cells. At 48–72 h post-transfection, the cells were harvested for analysis of genome modification efficiency using GeneArt Genomic Cleavage Detection kit. Alternatively, cells were analyzed by flow cytometry to determine the percentage of EmGFP positive cells.

For transfection of human iPSC, the cells were treated with TrypLE and plated onto Geltrex-coated 24-well plates at 40,000 cells per well, leading to approx. 30–40 % confluence at the time of transfection. One µg GeneArt Platinum Cas9 nuclease, 250 ng gRNA and 6 µl Cas9 Plus reagent were used to prepare the Cas9 RNPs instead, while the amount of Lipofectamine CRISPRMAX reagent remained constant at 1.5 µl. At around 6 h post-transfection, the media containing the transfection reagent was removed and replaced with fresh Essential 8 Medium. The cells were analyzed at 48 h post-transfection.

A ‘reverse’ transfection protocol was used to transfect MCF-7 and HepG2 cells. In this case, the Cas9 RNPs and Lipofectamine CRISPRMAX reagent were prepared in two separate tubes as described above with 500 ng GeneArt Platinum Cas9 nuclease, 125 ng gRNA, and 1 µl Cas9 Plus reagent in Tube-1, and 1.5 µl Lipofectamine CRISPRMAX reagent in Tube-2, respectively. The Cas9 RNPs solution was then transferred to the Lipofectamine CRISPRMAX solution. Upon vortexing, the mixture was incubated at 25 °C for 10–15 min to form the Cas9 RNPs and Lipofectamine CRISPRMAX complexes. Meanwhile, MCF-7 and HepG2 cells were detached with TrypLE and counted, followed by seeding at 2 × 10^5^ and 1.0 × 10^5^ cells per well, respectively. The Cas9 RNP/Lipofectamine CRISPRMAX solution was then added directly to the cell suspension and incubated for 48–72 h prior to analysis.

For Neon electroporation, adherent cells were detached from culture dishes and counted. In general, 10^5^ adherent cells or 2 × 10^5^ suspension cells were used per 10 µl reaction. For the Neon 24-well optimization protocol, 24 µg Cas9 protein and 6 µg gRNA were added to 120 µl Resuspension Buffer R, followed by mixing and incubation at 25 °C for 5 min to form the Cas9 RNPs. 2.4 × 10^6^ adherent cells or 4.8 × 10^6^ suspension cells were harvested and washed with DPBS. After aspiration, the cell pellets were re-suspended in 120 µl Resuspension Buffer R and then mixed with Cas9 RNPs. A 10 µl sample was taken for each electroporation using one of the Neon 24-well optimization conditions. The electroporated cells were transferred immediately to a 24 well containing 500 µl corresponding growth medium and incubated for 48 h prior to analysis. Upon optimization, the use of a higher dose of Cas9 RNPs (for example, 2 µg Cas9 protein and 500 ng gRNA per reaction) could further increase the cleavage efficiency (Liang et al. 2015).

### Genomic cleavage assay

The genomic modification efficiency was determined by GeneArt Genomic Cleavage Detection kit as described in the manual. The targeting gRNA sequence: 5′-gcatttctcagtcctaaaca**ggg**-3′ was used to edit HPRT1 locus. At 48 h post-transfection, suspension cells were harvested by centrifugation and then washed with DPBS, whereas adherent cells were washed directly with DPBS. Cells were lysed with 20 µl cell lysis buffer per 96-well or 50 µl cell lysis buffer per 24-well. Upon treatment with Proteinase K at 68 °C for 15 min, the mixture was held at 95 °C for 10 min. One to 3 µl of cell lysate was then used for PCR amplification with AmpliTaq Gold 360 Master Mix in the presence of the corresponding forward and reverse primers. For the HPRT1 target, a forward primer: 5′-acatcagcagctgttctg-3′ and a reverse primer: 5′- ggctgaaaggagagaact-3′ were used. The PCR program was set at 95 °C for 3 min for one cycle, then at 95 °C for 30 s, 55 °C for 30 s, and 72 °C for 30 s for a total of 40 cycles. Final extension was set at 72 °C for 5 min. The resulting PCR product (3 µl) was mixed with 1 µl of 10x Detection Reaction Buffer and 5 µl water, and then subjected to denaturation and re-annealing at 95 °C for 5 min, 4 °C for 5 min, 37 °C for 5 min, and then 4 °C for 5 min. Finally, 1 μl 10× detection enzyme was added to the sample and then incubated at 37 °C for 1 h. The digested product was analyzed with a 2 % E-gel EX agarose. The percentage of cleavage was quantified using an AlphaImager gel documentation system running AlphaView, Version 3.4.0.0. ProteinSimple (San Jose, CA, USA).

### Generation of a disrupted EmGFP stable cell line for homologous recombination assay

GripTite HEK293 stable cells expressing EmGFP were prepared via the Jump-In system as described in the manual (Thermo Fisher Scientific). To generate a disrupted EmGFP mutant stable cell line, 1.5 µg of GeneArt Platinum Cas9 nuclease was associated with 300 ng gRNA targeting the 5′-ctcgtgaccaccttcaccta**cgg**-3′ sequence in the EmGFP reporter gene (T1) and were then transfected into wild type EmGFP cells via electroporation, followed by limiting dilution to isolate clonal cell lines. Upon DNA sequencing, a disrupted EmGFP stable cell line with the 5′-CACCTT-3′ deletion was selected for a homologous recombination assay (Supplementary Table 1).

To create homologous recombination assays, a gRNA targeting the 5′-gaagcactgcacgccgtaggtgg-3′sequence within the disrupted EmGFP reporter gene (T2) was designed and synthesized. This gRNA only recognized the disrupted EmGFP gene but not the wild type EmGFP gene. One day prior to transfection, the cells were seeded on a 24 well plate at 10^5^ per well. 500 ng of GeneArt Platinum Cas9 nuclease and 125 ng gRNA were transfected into the disrupted EmGFP stable cell line using 1.5 µl Lipofectamine CRISPRMAX in the presence or absence of 500 ng of a 97 bp single-stranded DNA oligonucleotide (5′-catgtggtcggggtagcgggcgaagcactgcacgccgtaggtgaaggtggtcacgagggtgggccagggcacgggcagcttgccggtggtgcagatg-3′) or 300 ng of a 400 bp linear wild type PCR fragment amplified using a forward primer 5′-atggtgagcaagggcgaggagctg-3′ and a reverse primer 5′-gtcctccttgaagtcgatgccc-3′ (Supplementary Table 2). At 48 h post-transfection, the restoration of EmGFP function was determined by flow cytometric analysis with an Attune NxT Acoustic Focusing Cytometer (Thermo Fisher Scientific).

## Results and discussion

### Identification of Lipofectamine CRISPRMAX

**Fig. 1:**
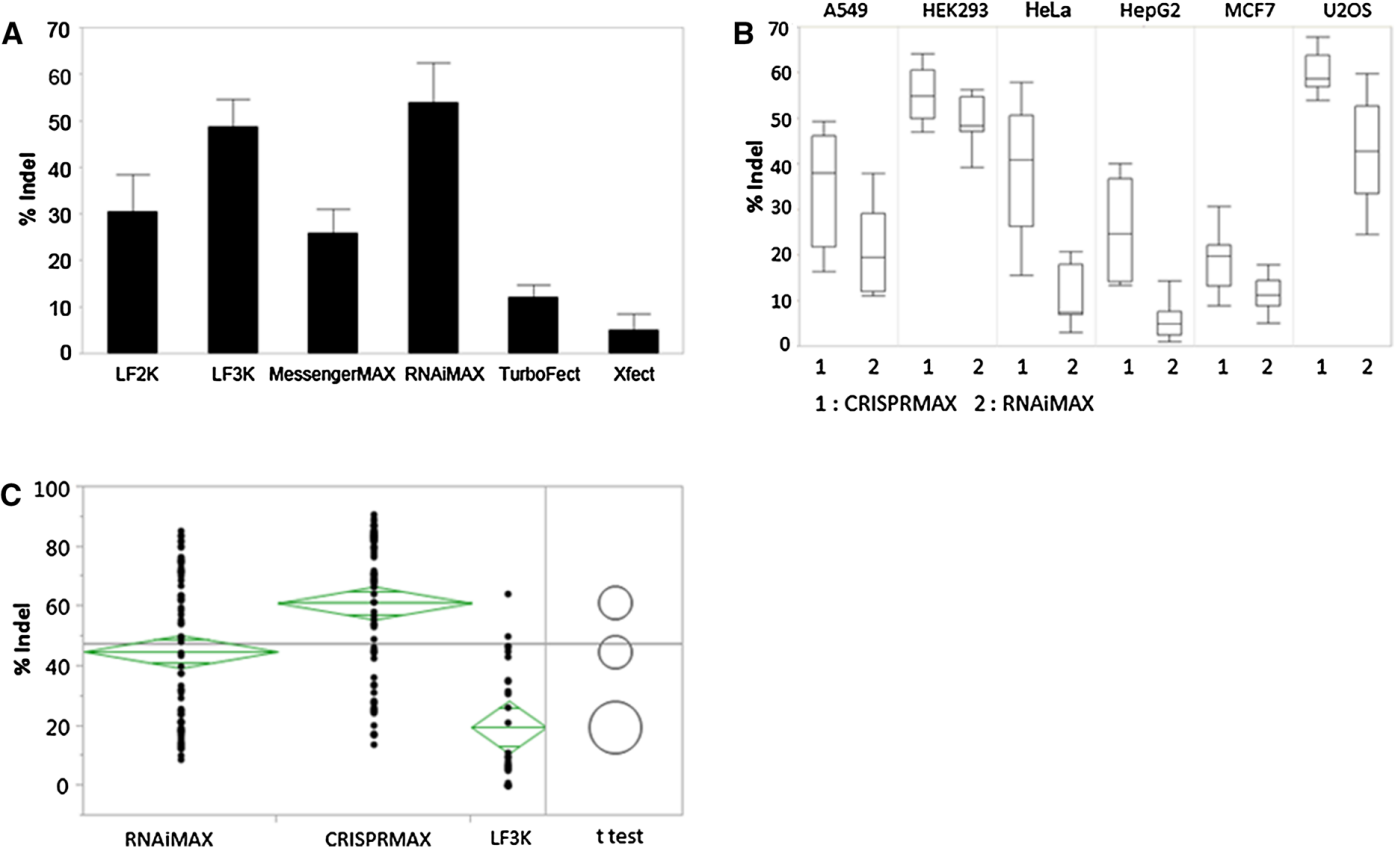
Identification of Lipofectamine CRISPRMAX**. a** One day prior to transfection, HEK293 and HCT116 were seeded on a 24 well plate at 10^5^ cells per well. On the day of transfection, 500 ng Cas9 protein and 120 ng HPRT1 gRNA were transfected with either Lipofectamine 2000 (LF2K), Lipofectamine 3000 (LF3K), Lipofectamine MessengerMAX, Lipofectamine RNAiMAX, TurboFect or Xfect transfection reagent according to manufacturer’s protocol. At 48 h post-transfection, cells were harvested to perform genomic cleavage assays. The % of Indel was quantified using AlphaView software. The data was analyzed using JMP statistical software. The *bar graph* represented the mean and standard deviation of three independent experiments of two cell lines. **b**, **c** A549, HEK293, HepG2, HeLa, MCF-7 and U2OS were seeded on 96-well plates at approx. 20,000 cells per well and then transfected with 40 ng Cas9 protein and 8.5 ng HPRT gRNA using either Lipofectamine CRISPRMAX, Lipofectamine RNAiMAX or Lipofectamine 3000. After 48 h post-transfection, the cells were lysed and subjected to a genomic cleavage assay. The results were processed using JMP11 software. **b** was the Box Plot of Indel frequencies comparing Lipofectamine CRISPRMAX to Lipofectamine RNAiMAX in six different cell lines. **c** was one-way ANOVA analysis of % Indel by type of lipid with blocking of cell type. Each *dot* represented one experimental data point. The *diamond* represented the 95 % confidence interval for the mean of each group. The *circles* were visual representations of group mean comparisons using Student’s *t*-tests. The *p* value was less than 0.05

To identify the best transfection reagents for delivery of Cas9 RNPs, initially we compared several commercially available protein transfection reagents, including Lipofectamine 2000, Lipofectamine 3000, Lipofectamine RNAiMAX, Lipofectamine MeassengerMax, TurboFect, and Xfect Protein transfection reagent. Cas9 protein and HPRT1 gRNA were transfected into HEK293 and HCT116 cell lines according to manufacturer’s protocol. Upon 48 h post-transfection, the cells were harvested to analyze the genome modification efficiencies. As depicted in Fig. 1a, the Indel (insertions and deletions) production efficiencies were highest when using Lipofectamine 3000 or Lipofectamine RNAiMAX transfection reagents. Next, we used Lipofectamine 3000 and Lipofectamine RNAiMAX as controls and screened more than 60 transfection reagents. Screening was conducted in a 96-well format using three easy-to-transfect cell lines (HEK293, HeLa, and U2OS) and three hard-to-transfect cell lines (HepG2, A549, and MCF-7). Cells were seeded on 96-well plates 1 day prior to transfection. On the day of transfection, the Cas9 protein was incubated with HPRT1 gRNA at 25 °C for 5 min in Opti-MEM medium to form the Cas9 RNPs, followed by mixing with various amount of transfection reagents in Opti-MEM medium. The percentage of Indels was determined using the GeneArt genomic cleavage detection assay at 48 h post-transfection. Among all the formulations, only a few worked equally well or better than Lipofectamine RNAiMAX with one formulation (Lipofectamine CRISPRMAX) standing out.

As shown in Fig. 1b, the percentage of Indels in cells transfected with Lipofectamine CRISPRMAX was higher than those with Lipofectamine RNAiMAX, especially in A549, HeLa and HepG2 cell lines. Based on statistical analysis, the genome cleavage efficiencies in Lipofectamine CRISPRMAX-transfected cell lines were significantly higher than those in Lipofectamine 3000 or RNAiMAX-transfected cell lines, with an average increase of 40 or 15 %, respectively, across six different cell lines (Fig. 1c). The visual comparison of group means using Student’s *t*-tests, represented by the circles, showed that Lipofectamine CRISPRMAX, Lipofectamine 3000 and RNAiMAX were significantly different from each other as the circles did not intersect.

### Time course of Cas9 RNPs activity

**Fig. 2:**
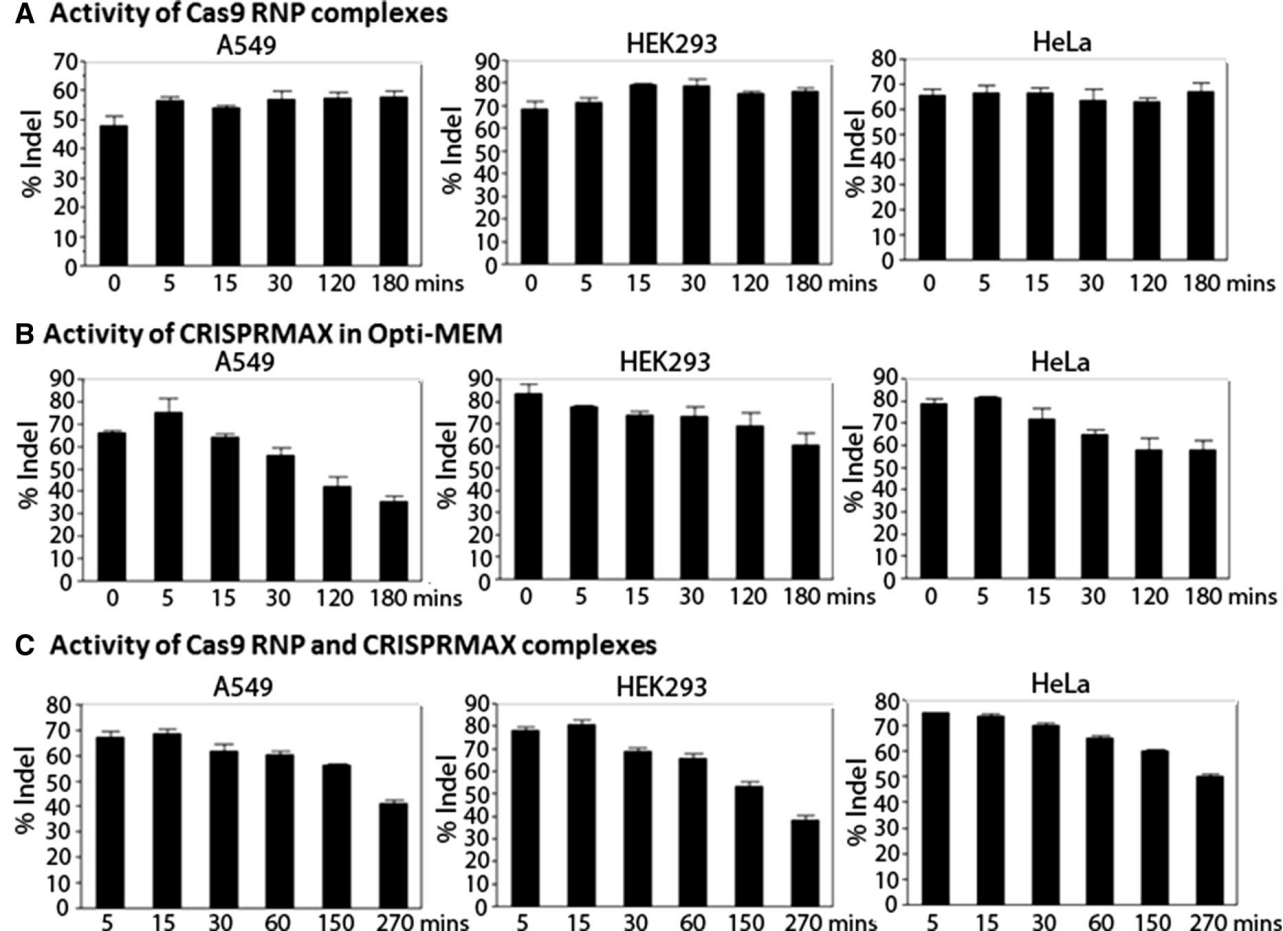
Activities of Cas9 RNP complexes and Cas9 RNP/Lipofectamine CRISPRMAX complexes. One day prior to transfection, cells were seeded on a 96 well plates. A master mixes of Cas9 RNP complexes and Lipofectamine CRISPRMAX were prepared in Opti-MEM media based upon 40 ng Cas9 protein, 8.5 ng gRNA, and 0.3 µl Lipofectamine CRISPRMAX per well. The incubation times of Cas9 RNP (**a**), Lipofectamine CRISPRMAX (**b**), and Cas9 RNP/Lipofectamine CRISPRMAX complexes (**c**) served as dependent variables. At the indicated time point, aliquots of Cas9 RNP complexes in Opti-MEM were added to aliquots of Lipofectamine CRISPRMAX solution and incubated for indicated time prior to addition to A549, HEK293, and HeLa cells, respectively. Upon 48 h post-transfection, the genome cleavage efficiencies were determined

Upon identification of Lipofectamine CRISPRMAX as the best transfection reagent, we determined the functional activity of Cas9 RNPs by examining the times required for complexation of Cas9 protein with gRNA (Cas9 RNPs) and Cas9 RNPs with Lipofectamine CRISPRMAX in A549, HEK293, and HeLa cells. As shown in Fig. 2a, the Cas9 RNPs remained active at 25 °C for up to 3 h based on the genome cleavage assay. On the other hand, the diluted Lipofectamine CRISPRMAX were only active at 25 °C for 5–15 min as a longer incubation time had a decreased cleavage efficiency (Fig. 2b). When the Cas9 RNPs were mixed with Lipofectamine CRISPRMAX the editing efficiency dropped after 10–15 min indicating that the addition of Lipofectamine CRISPRMAX was the limiting step (Fig. 2c).

### Main factors regulating transfection efficiency

**Fig. 3:**
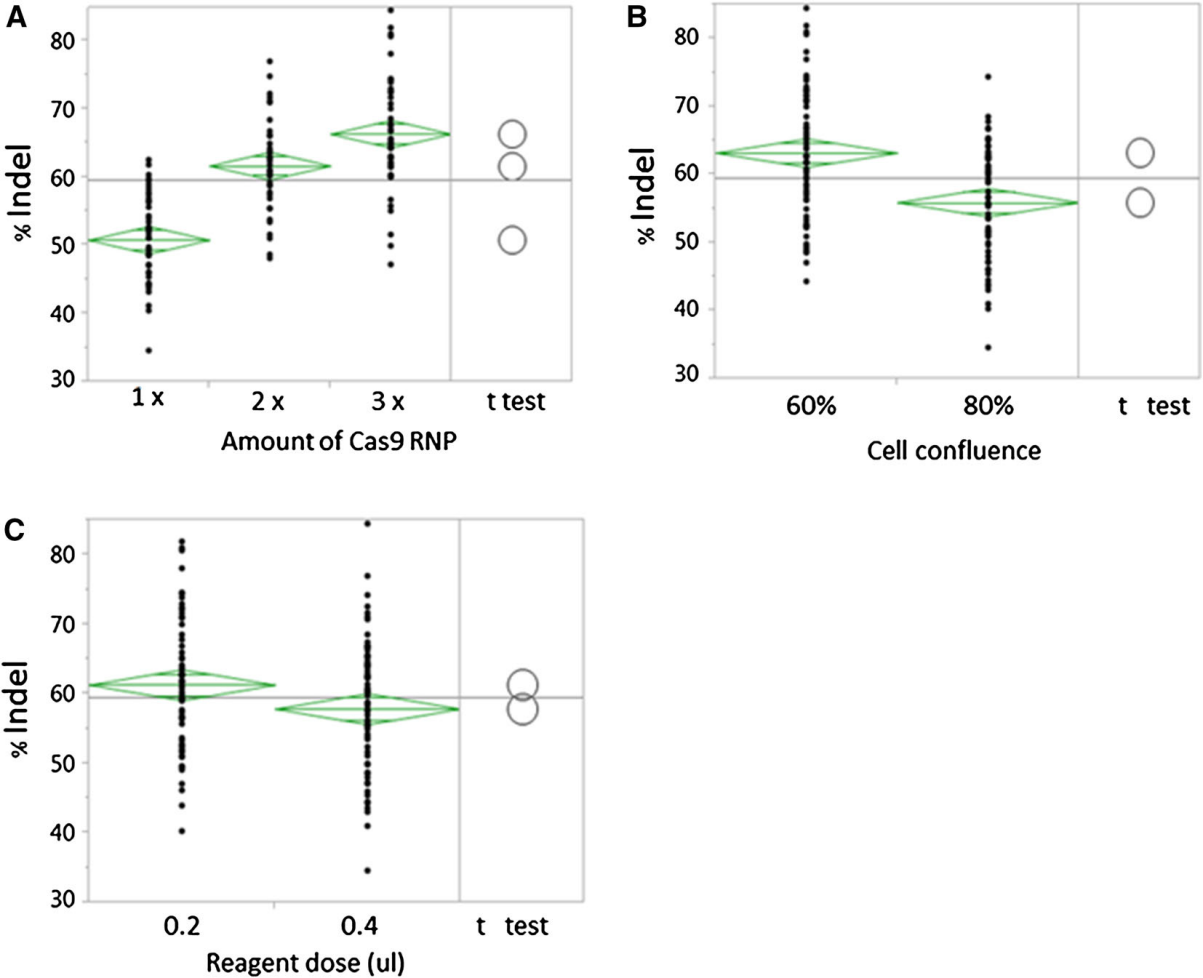
Factors regulating transfection efficiencies. **a** A549, HEK293, HepG2, HeLa, MCF-7 and U2OS were seeded on 96-well plates at two cell densities and then transfected with either 40 ng Cas9 protein and 8.5 ng gRNA (1×), 80 ng Cas9 protein and 17 ng gRNA (2×) or 120 ng Cas9 protein and 25.5 ng gRNA (3×) using either 0.2 or 0.4 µl Lipofectamine CRISPRMAX. The editing efficiency was determined at 48 h post-transfection. The Indel percentage was determined using the AlphaView software and the resulting data was processed using JMP11 software. Analysis of variance (ANOVA) analysis of % Indel by dose of Cas9 RNP (**a**), cell density (**b**), and amount of transfection reagent (**c**) were carried out across six different cell lines. Each *dot* represented one data point, whereas the *diamond* represented the 95 % confidence interval. The *circles* were visual views of group mean comparisons using Student’s *t*-tests. The *p* value was less than 0.05

Next, we examined the key factors that governed the transfection efficiency by varying the dose of transfection reagent, the amount of Cas9 RNPs, and cell density. Six commonly used cell lines were transfected with increasing amount of Cas9 RNPs, followed by genome cleavage assay. As shown in Fig. 3a, the efficiency of Indel production in transfected cells increased with an increase of Cas9 RNPs. The differences between the doses were statistically significant because the group mean comparisons represented by the circles did not intersect and the *p* value was <0.05. To transfect cells in a 96-well plate, the optimal amounts of Cas9 protein and gRNA were approx. 120 and 25.5 ng respectively. Cell seeding density plays an important role in regulating the transfection efficiency. As depicted in Fig. 3b, the average genome modification efficiency across six different cell lines was significantly higher at 60 % cell confluence than at 80 % cell confluence at the time of transfection with a *p* value less than 0.05. However, no significant difference in editing efficiency was observed between low and high lipid doses (Fig. 3c). Other factors, such as cell passage and dissociation, also contributed to daily variation in cell transfection and Indel efficiency.

### Low cell toxicity of Lipofectamine CRISPRMAX

**Fig. 4:**
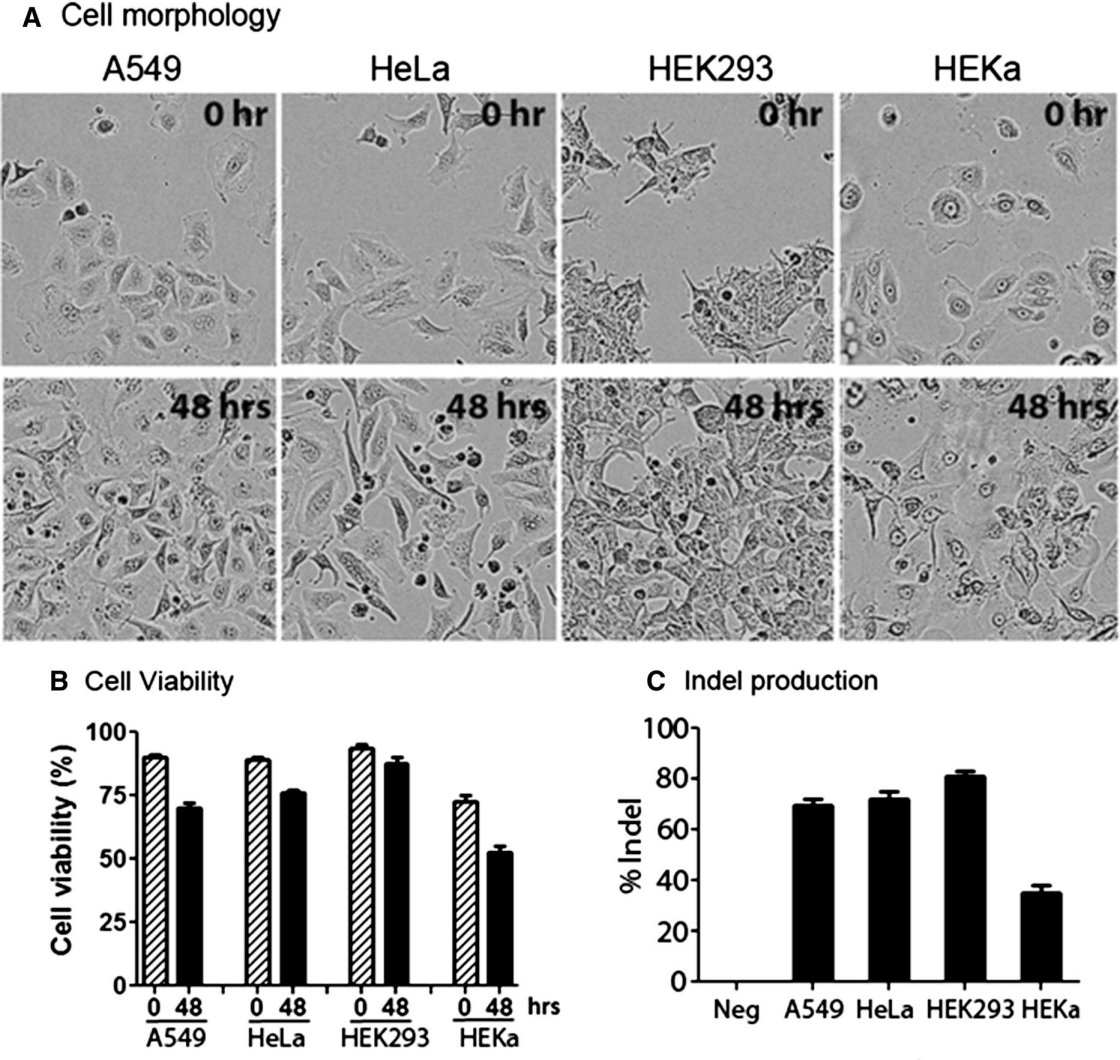
Cell toxicity using Lipofectamine CRISPRMAX. **a** Prior to transfection and at 48 h post-transfection, the morphologies of A549, HeLa, HEK293, and human epidermal keratinocytes (HEKa) were examined by an IncuCyte instrument, Essen BioScience Inc. (Ann Arbor, MI, USA). **b** Cell viabilities were measured by Trypan Blue staining before 0 and after 48 h post-transfection. **c** The genome modification efficiencies were determined at 48 h post-transfection

We then scaled up to 24 wells to test a set of 23 cell lines, including a variety of adherent and suspension cells from different species. The morphologies of more than a dozen adherent cell lines were recorded prior to transfection and at 48 h post-transfection (Supplementary Fig. 1). Most of the cells looked healthy under the microscope with examples shown in Fig. 4a, very little floating dead cells were observed upon 48 h post-transfection for A549, HeLa, HEK293, and human epidermal keratinocytes (HEKa). Cell viability assays with Trypan Blue indicated that the viable cells only decreased moderately after transfection compared to control cells, suggesting that the cell toxicity induced by Lipofectamine CRISPRMAX was relatively low (Fig. 4b). We observed around 68, 71, 80, and 35 % genome cleavage efficiencies in A549, HeLa, HEK293, and HEKa primary cell lines, respectively (Fig. 4c). The low cell toxicity of Lipofectamine CRISPRMAX prompted us to transfect cells at much lower cell density so as to increase the transfection efficiency (Table 1 and Supplementary Table 3). For example, N2A, mouse ESC and iPSC were grown to 35, 25 and 30 % confluence at the time of transfection (Supplementary Table 3) and achieved 70, 75 and 55 % genome editing efficiencies at mouse Rosa26 and human HPRT1 loci, respectively (Table 1). The improved efficiencies were probably due to the higher accessibility of transfection reagents at low cell density. However, the optimal cell density was highly dependent on cell type and needed to be determined experimentally.

**Table 1:** Genome editing efficiency in a variety of cell lines

	Cell line	Source	Lipofectamine CRISPRMAX (% Indel)	Neon electroporation (% Indel)
1	mESC	Mouse embryonic stem cell	75 ± 3	74 ± 4
2	N2A	Mouse liver carcinoma	70 ± 5	81 ± 2
3	3T3	Mouse embryonic fibroblast	57 ± 4	50 ± 2
4	CHO	Hamster ovary	57 ± 1	–
5	COS-7	Monkey kidney	44 ± 3	–
6	A549	Human lung carcinoma	48 ± 3	66 ± 3
7	293FT	Human kidney	85 ± 5	88 ± 3
8	HEK293	Human kidney	75 ± 5	–
9	HCT116	Human colon carcinoma	85 ± 5	–
10	HEKa	Human primary epidermal keratinocytes	14 ± 2	32 ± 2
11	HeLa	Human cervical cancer	50 ± 7	–
12	HepG2	Human liver cancer	30 ± 3	52 ± 3
13	HUVEC	Human umbilical vein endothelium	9 ± 3	26 ± 2
14	iPSC	Human induced pluripotent stem cell	55 ± 3	85 ± 2
15	MCF-7	Human mammary gland	8 ± 4	22 ± 5
16	MDA-MB-231	Human breast cancer	39 ± 5	–
17	U2OS	Human osteosarcoma	55 ± 4	70 ± 3
18	Jurkat	Human T cell leukemia	19 ± 3	94 ± 2
19	K562	Human lymphoblastoid	20 ± 2	91 ± 1
20	THP-1	Human monocytes	12 ± 3	31 ± 3
21	SC-1	Human B lymphoblasts	0	44 ± 2
22	Raji	Human B lymphocyte	0	50 ± 5
23	NK-92	Human peripheral blood	0	31 ± 5

Mouse Rosa26, human HPRT1, monkey Nr0b1, and hamster COSMC loci were selected for genome editing. The average and standard deviation from Neon electroporation were calculated based on the top three protocols. Detailed electroporation protocol was described in Table 4s

– Not tested

### Comparison of Lipofectamine CRISPRMAX to electroporation

Suspension cells, especially hematopoietic cells, are difficult to transfect by conventional lipid reagents (Papapetrou et al. 2005). We also found that hematopoietic cells were hard to transfect using Lipofectamine 3000, Lipofectamine RNAiMAX, and Lipofectamine CRISPRMAX. For each hard-to-transfect cell line, we tested the delivery of Cas9 RNPs using the Neon 24-well optimization protocol (Supplementary Table 4). For example, using electroporation we achieved 94, 91 and 44 % Indel production efficiencies in Jurkat T cells, K562 and SC-1 cells respectively at the HPRT1 locus, whereas relatively low genome modification efficiencies were observed using Lipofectamine CRISPRMAX in these suspension cell lines (Table 1).

### Precise genome editing using Lipofectamine CRISPRMAX

**Fig. 5:**
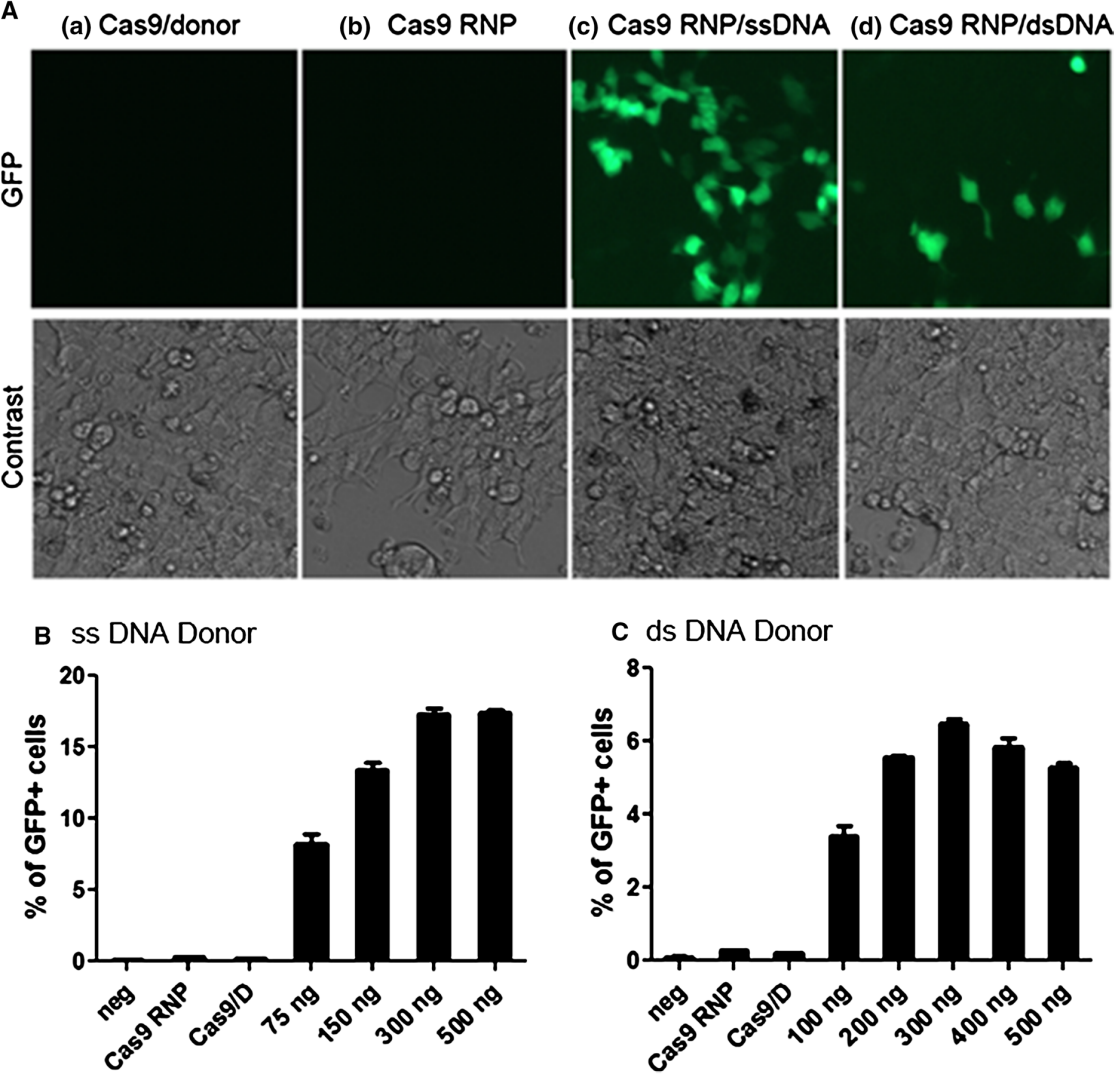
Co-delivery of Cas9 RNP and donor DNA. Various amount of a 97 bp single-stranded DNA oligonucleotide (ssDNA) or a 400 bp double-stranded DNA fragment (dsDNA) was co-delivered with Cas9 RNPs into a disrupted EmGFP stable cell line in 24-well culture plates. After 48 h post-transfection, the percentages of GFP positive cells were quantified using flow cytometric analysis. Delivery of Cas9 RNP or Cas9 plus donor DNA (Cas9/D) served as controls. The experiments were performed in triplicate

Precise gene modification, such as single-nucleotide polymorphism (SNP) correction in cancer cells, is an important aspect in biomedical and clinical applications (Lee 2012). For a proof of concept, we tested the co-delivery of various amount of donor DNA with Cas9 RNPs into a stable GripTite HEK293 cell line harboring a disrupted EmGFP gene using Lipofectamine CRISPRMAX. Delivery of Cas9 RNPs alone or Cas9 protein plus donor DNA was used as controls. After 48 h post-transfection, the cells were examined by a fluorescence microscope and images were recorded (Fig. 5a). The transfected cells were subjected to flow cytometric analysis to determine the percentage of EmGFP positive cells. As shown in Fig. 5b, approx. 17 % of the cells restored the function of EmGFP when 500 ng 97 bp ssDNA oligonucleotide (ssODN) was used, whereas approx. 6.5 % of EmGFP positive cells were observed when 300 ng of a 400 bp dsDNA fragment was used. The homologous recombination efficiency we obtained is comparable to those observed by other research groups. For example, the delivery of Cas9 RNPs and ssODN into unsynchronized HEK293T cells via nucleofection yielded around 10 % HDR frequencies, although up to 38 % HDR efficiency was observed in synchronized cells (Lin et al. 2014). The delivery of Cas9 RNPs and ssODN into an EGFP-repair reporter cell line using Lipofectamine 2000 resulted in 8–11 % of HDR frequencies (Zuris et al. 2015). Although the HDR efficiency is getting better, there is undoubtedly room for further improvement.

## Conclusion

Lipofectamine CRISPRMAX is more robust than Lipofectamine RNAiMAX and several other transfection reagents in delivery of Cas9 protein/gRNA complexes into a variety of cell lines. However, delivery of the Cas9 RNPs by Neon electroporation was effective across all different cell lines tested and often was the only method that could produce Indels in difficult to transfect suspension cell lines. Because of the ease of use and low toxicity, Lipofectamine CRISPRMAX will further facilitate high throughput drug screening and genome editing where electroporation is less applicable.

## Electronic supplementary material

Below is the link to the electronic supplementary material.

Supplementary material 1 (PDF 191 kb)
