# Supplementary material for: Improved delivery of Cas9 protein/gRNA complexes using lipofectamine CRISPRMAX
Source: Biotechnol Lett. 2016 Feb 18;38:919–29. doi: 10.1007/s10529-016-2064-9 (PMC4853464; doi:10.1007/s10529-016-2064-9)
Supplement: Supplementary file 1 — Supplementary material 1 (PDF 191 kb) [file 10529_2016_2064_MOESM1_ESM.pdf]

Supplemental Material

Materials

Gibco® Human Episomal iPSC, Human Epidermal Keratinocytes, Mouse Embryonic Fibroblasts (MEF), mouse ESC, 293FT cells, HUVEC, DMEM, RPMI 1640, IMDM, DMEM/F-12, Knockout™ DMEM, Non-essential amino acids solution, recombinant human LIF, Knockout™ Serum Replacement, McCoy's, Medium 200, EpiLife® Medium, Essential 8™ Medium, Fetal Bovine Serum (FBS), HKGS kit, Glutamax™, LSGS Kit, TrypLE™ Express Enzyme, Geltrex®, Opti-MEM® Medium, FluoroBrite™ DMEM, Lipofectamine® 2000, Lipofectamine® 3000, Lipofectamine® RNAiMAX™, Lipofectamine® MessengerMAX™, Lipofectamine® CRISPRMAX™, TurboFect, Jump-In™ GripTite™ HEK293 cells Retargeting Kit, Neon® Transfection System 10 µL Kit, pJTI R4 EXP CMV EmGFP pA, 2% E-Gel® EX Agarose Gels, TranscriptAid T7 High Yield Transcription Kit, MEGAclear™ Transcription Clean-Up Kit, Qubit® RNA BR Assay Kit, GeneArt® Genomic Cleavage Detection Kit, and GeneArt® Platinum™ Cas9 Nuclease, and oligonucleotides were from Thermo Fisher Scientific. Jurkat T cells, K562 cells, 3T3, COS-7, CHO-S, N2A, A549, HEK293, HeLa, MCF-7, MDA-MB-231, U2OS, HepG2, SC-1, HCT116, NK-92, THP-1, and Raji cell lines were obtained from American Type Culture Collection (ATCC). Xfect™ Protein transfection reagent was purchased from Clontech Laboratories, Inc. (Palo Alto, CA, USA). Guide RNAs (gRNAs) were synthesized via one-pot PCR assembly of a gRNA template followed by *in vitro* transcription (9). CRISPR target sequences and primer sequences for amplification of the genomic locus are described in Supplementary Table S2.

Cell culture

HEK293, 3T3, HeLa, MCF-7, MDA-MB-231, HepG2, COS-7, N2A, and A549 were grown in DMEM medium supplemented with 10% FBS. HCT116 and U2OS were cultured in McCoy's medium containing 10% FBS, while CHO cells were maintained in DMEM/F-12 plus 10% FBS. HUVEC cells were propagated in Medium 200 supplemented with LSGS. Human Epidermal Keratinocytes (HEKa) were maintained in EpiLife® Medium supplemented with HKGS. Jurkat T cells, SC-1, THP-1, and Raji cells were grown in RPMI medium supplemented with 10% FBS, whereas K562 cells were maintained in IMDM medium containing 10% FBS. NK92 cells were grown on RPMI medium supplemented with 25% FBS, 1 mM sodium pyruvate, 10 mM HEPES and 0.1 mM β-mercaptoethanol. Mouse ESCs were cultured on MEF feeder layers using Knockout™ DMEM supplemented with 15% knockout™ Serum Replacement, non-essential amino acids, L-glutamine, β-mercaptoethanol and 10 ng/ml recombinant human LIF. The Gibco® iPSC line was maintained in Essential 8™ Medium on Geltrex® matrix-coated culture vessels and passaged using 0.5 mM EDTA prepared in Dulbecco's Phosphate-Buffered Saline (DPBS) without calcium or magnesium. All the cells were incubated at 37°C in a humidified 5% CO_2 incubator.

Supplementary Table 1. EmGFP sequence.

atggtgagcaagggcgaggagctgttcaccggggtggtgcccatcctggtcgagctggacgg
cgacgtaaacggccacaagttcagcgtgtccggcgagggcgagggcgatgccacctacggc
aagctgaccctgaagttcatctgcaccaccggcaagctgcccgtgccctggcccaccctcgtg
ac~~cacctt~~cacctacggcgtgcagtgcttcgcccgctaccccgaccacatgaagcagcacgac
ttcttcaagtccgccatgcccgaaggctacgtccaggagcgcaccatcttcttcaaggacgacg
gcaactacaagacccgcgccgaggtgaagttcgagggcgacaccctggtgaaccgcatcga
gctgaagggcatcgacttcaaggaggacggcaacatcctggggcacaagctggagtacaac
tacaacagccacaaggtctatatcaccgccgacaagcagaagaacggcatcaaggtgaactt
caagacccgccacaacatcgaggacggcagcgtgcagctcgccgaccactaccagcagaac
acccccatcggcgacggccccgtgctgctgcccgacaaccactacctgagcacccagtccgcc
ctgagcaaagaccccaacgagaagcgcgatcacatggtcctgctggagttcgtgaccgccgc
cgggatcactctcggcatggacgagctgtacaagtaa

Note: Six nucleotides “cacctt” with strikethrough are deleted in mutant cell line. The reverse sequence (5’-gaagcactgcacgccgtaggtgg-3’) of Red-highlighted sequence serves as a new CRISPR target for homologous recombination assay. Sequences highlighted in Green were used for PCR amplification of a ds donor DNA fragment.

Supplementary Table 2. CRISPR target sequences and PCR primers.

Species	Locus	target Sequence (5'->3')	Forward primer (5'->3')	Reverse primer (5'->3')
Human	HPRT1	gcatttctcagtcctaaacaggg	acatcagcagctgttctg	ggctgaaaggagagaact
Hamster	COSMC	gaatatgtgagtgtggatggagg	ggatccatcgcagcctttct	actacctggttcgggtggtt
Monkey	Nr0b1	ggcgctcaagagtccacaggtgg	agcatcctctacagcttgctcac	ggtactgatgttcagactccagc
Mouse	Rosa26	agatgggcgggagtcttctggg	gggctgagcggctgcggggcg	ctgtagtaaggatctcaagcaggag
EmGFP	EmGFP-T1	ctcgtgaccaccttcacctacgg	atggtgagcaagggcgaggagctg	gtcctccttgaagtcgatgccc
EmGFP	EmGFP-T2	gaagcactgcacgccgtaggtgg	atggtgagcaagggcgaggagctg	gtcctccttgaagtcgatgccc

Supplementary Table 3. Genome editing efficiency in a variety of cell lines (24-well Format)

	Cell line	Cell Seeding Numbers/well (x10^3)	Confluence prior to transfection (%)	Dosage: Cas9/gRNA (ng/ng)	Dosage: Cas9 PLUS™ Reagent (µl)	Dosage: Lipofectamine® CRISPRMAX™ (µl)	Lipofectamine® CRISPRMAX™ (% Indel)	Neon Electroporation (10ul tips): Protocol (Program #)	Neon Electroporation (10ul tips): Cell Numbers (x10^3)	Neon Electroporation (10ul tips): Cas9/gRNA (ng/ng)	Neon Electroporation (10ul tips): % Indel
1	mouse ESC	100	25	500/125	1	2	75 ± 3	1600v/10ms/3pulses(#24)	150	1000/250	74 ± 4
2	N2A	42	35	500/125	1	1.5	70 ± 5	1400v/30ms/1pulse(#9)	100	1000/250	81 ± 2
3	3T3	45	43	500/125	1	1.5	57 ± 4	1600v/10ms/3pulses(#24)	120	1000/250	50 ± 2
4	CHO	60	30	500/125	1	1.5	57 ± 1	-	-	-	-
5	COS-7	90	75	500/125	1	1.5	44 ± 3	-	-	-	-
6	A549	60	65	500/125	1	1.5	48 ± 3	1200v/20ms/4pulses(*)	120	1000/250	66 ± 3
7	293FT	90	50	500/125	1	2	85 ± 5	1150v/20ms/2pulses(*)	150	1000/250	88 ± 3
8	HEK293	120	45	500/125	1	1.5	75 ± 5	-	-	-	-
9	HCT116	90	49	500/125	1	1.5	85 ± 5	-	-	-	-
10	HEKa	90	60	500/125	1	1.5	14 ± 2	1400v/20ms/2pulses(#16)	100	2000/500	32 ± 2
11	HeLa	60	55	500/125	1	1.5	50 ± 7	-	-	-	-
12	HepG2	90	40	500/125	1	1.5	30 ± 3	1300v/30ms/1pulse(#8)	120	1000/250	52 ± 3
13	HUVEC	90	70	500/125	1	1.5	9 ± 3	1600v/10ms/3pulses(#24)	100	2000/500	26 ± 2
14	iPSC	40	30	1000/250	6	1.5	55 ± 3	1200v/20ms/2pulses(#14)	80	2000/500	85 ± 2
15	MCF-7	144	55	500/125	1	1.5	8 ± 4	1150v/30ms/2pulses(#20)	120	1000/250	22 ± 5
16	MDA-MB-231	90	45	500/125	1	1.5	39 ± 5	-	-	-	-
17	U2OS	90	80	500/125	1	1.5	55 ± 4	1400v/15ms/4pulses(*)	150	1000/250	70 ± 3
18	Jurkat	100	n/a	500/125	1	1.5	19 ± 3	1700v/20ms/1pulse(#5)	200	1500/350	94 ± 2
19	K562	100	n/a	500/125	1	1.5	20 ± 2	1400v/10ms/3pulses(#22)	200	1000/250	91 ± 1
20	THP-1	100	n/a	500/125	1	1.5	12 ± 3	1600v/10ms/3pulses(#24)	200	1000/250	31 ± 3
21	SC-1	100	n/a	500/125	1	1.5	0	950v/30ms/2pulses(#18)	200	1000/250	44 ± 2
22	Raji	100	n/a	500/125	1	1.5	0	1600v/10ms/3pulses(#24)	200	1000/250	50 ± 5
23	NK-92	100	n/a	500/125	1	1.5	0	1400v/10ms/3pulses(#22)	200	2000/500	31 ± 5

Mouse Rosa26, human HPRT1, monkey Nr0b1, and hamster COSMC loci were selected for genome editing. The average and standard deviation from Neon™ electroporation were calculated based on the top three protocols. (-) not tested. n/a: non-applicable. (*) special program, not included in Neon 24 conditions.

Supplementary Table 4. Neon optimization protocols

Protocol	1	2	3	4	5	6	7	8	9	10	11	12	13	14	15	16	17	18	19	20	21	22	23	24
Pulse Voltage (V)	0	1400	1500	1600	1700	1100	1200	1300	1400	1000	1100	1200	1100	1200	1300	1400	850	950	1050	1150	1300	1400	1500	1600
Pulse Width (ms)	0	20	20	20	20	30	30	30	30	40	40	40	20	20	20	20	30	30	30	30	10	10	10	10
# of Pulse	0	1	1	1	1	1	1	1	1	1	1	1	2	2	2	2	2	2	2	2	3	3	3	3

Supplementary Figure 1. Cell morphology

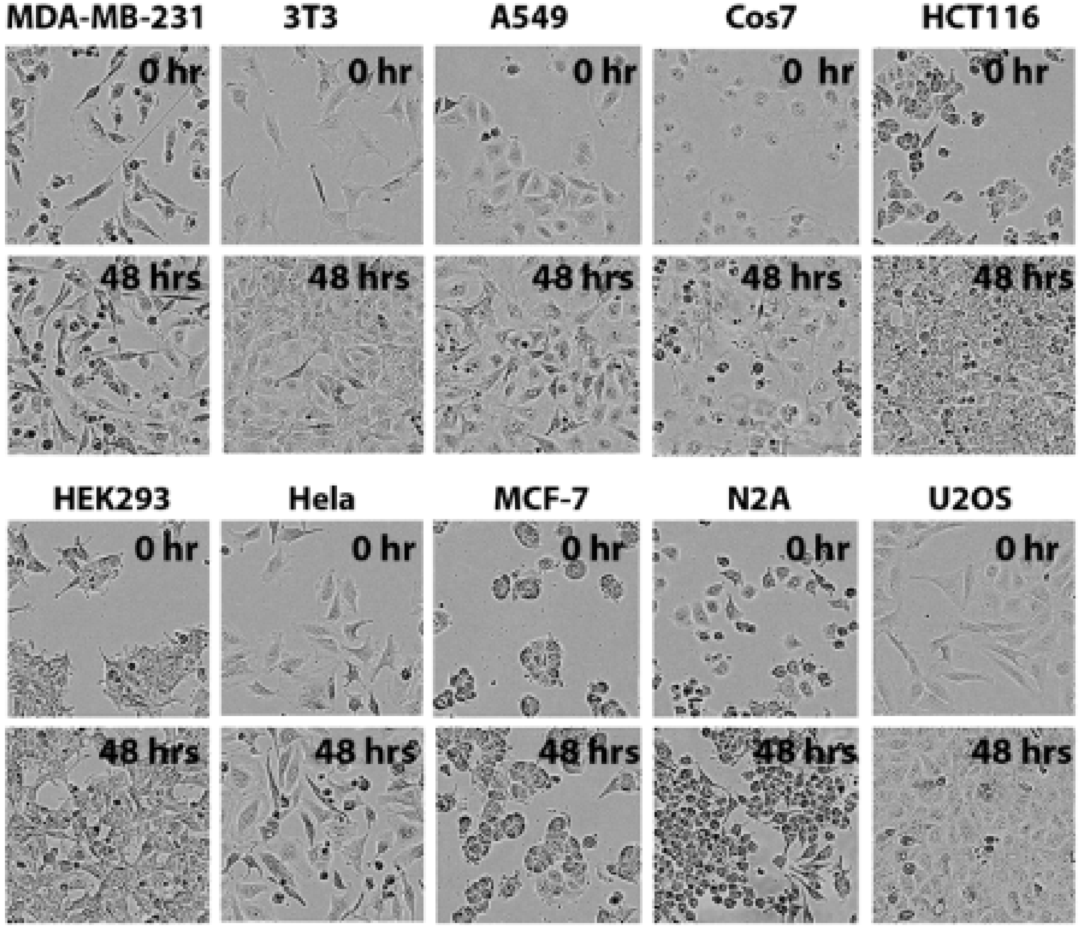

(Prior to cell transfection or at 48 hours post transfection, the cell morphologies were recorded using an IncuCyte instrument)
